# The complete chloroplast genome of a moss Korea *Climacium dendroides* (Hedw.) F. Weber & D. Mohr

**DOI:** 10.1080/23802359.2020.1731362

**Published:** 2020-02-28

**Authors:** Yeong-Deok Han, Youngeun Choi, SeungJin Park, Yong-Su Park, Young-Jun Yoon

**Affiliations:** a Research Center for Endangered Species, National Institute of Ecology, Yeongyang-gun, Korea;; b Department of Ecology Landscape Design, Chonbuk National University, Iksan, Korea;; c Department of Biology, Chonbuk National University, Jeonju, Korea

**Keywords:** *Climacium dendroides*, chloroplast genome, moss

## Abstract

The complete chloroplast (cp) genome sequence of *Climacium dendroides*, determined using Illumina sequencing data, is presented herein. The DNA sequence of *C. dendroides* is 124,957 bp in length and has an overall CG content of 29.0%, including 82 protein-coding genes, 36 transfer RNA genes, and 8 ribosomal RNA genes. Phylogenetic trees were constructed based on the cp genome sequences of 10 bryophytes downloaded from GenBank and one acquired from this study.

*Climacium dendroides* is a type of Bryophyta that belongs to the family Climaciaceae. The genus *Climacium* was characterized by Friedrich Weber & Daniel Matthias Heinrich Mohr (1804) in Naturhistorische Reise durch einen Theil Schwedens. *Climacium* is a small group of Hypnales, but it is a morphologically distinct genus (tree-mosses) with four species distributed mainly in the northern hemisphere (Jia et al. 2011). *Climacium* consists of four species, namely *C. americanum*, *C. dendroides*, *C. japonicum* and *C. kindbergii.* However, only three *Climacium* species, with the exception of *C*. *kindbergii,* have been identified in Korean moss flora (Choe 1980). Herein, we determined the complete chloroplast (cp) genome sequence of *Climacium dendroides* using Illumina sequencing data.

In this study, the cp genome sequence of *C. dendroides* (GenBank accession no.MT006132), as a representative of Climaciaceae, was sequenced and described. To date, the chloroplast genome of Climaciaceae species has not been reported in the literature. Chloroplast genome sequence of *C. dendroides* will be useful in the evolutionary studies of bryophytes.

Genomic DNA was extracted from a 5 × 5 cm area collected from the natural habitat of Jilmoen wetland, Odasan (37°45′59.6″N; 128°42′17.6″E) on 5 July 2019. The voucher specimen is stored at the Herbarium of Chonbuk National University (JNU Herbarium) in Korea with the accession number PSJ 20190705-1. Illumina paired-reads (2 × 300 bp) were employed for assembling the chloroplast genome of *C. dendroides* using Novoplasty 2.4 (Dierckxsens et al. 2016) and Geneious R8 8.1.9 (Biomatters Ltd., Auckland, New Zealand). Gene annotation was based on *Sanionia uncinata* using Geneious R8 8.1.9. A maximum-likelihood tree was constructed using MEGA7 (Kumar et al. 2016) based on the JTT matrix-based model, and using concatenated sequences of 16 protein-coding genes (*accD*, *atpA*, *atpB*, *cemA*, *chlB*, *chlL*, *clpP*, *ndhB*, *ndhF*, *ndhH*, *ndhK*, *petA*, *psaA*, *psbC*, *rbcL* and *rps2*) from 10 related species (7 Bryophyta, 1 Anthocerotophyta and 2 Marchantiophyta) (Kim et al. 2019; Cevallos et al. 2019).

The cp genome of *C. dendroides* was 124,957 bp in length and contained 82 PCGs, 36 transfer RNAs (tRNA), and 8 ribosomal RNAs (rRNA). This cp genome has two inverted repeats, each of 9692 bp, a large single-copy (87,030 bp) and small single-copy (18,543 bp). The total GC content of this cp genome is 29.0%, which is similar to that of *S. uncinata* (29.3%).

ML analysis showed that *C. dendroides* first formed a branch with *S. uncinata*, and then formed a single branch with Hypnales (Figure 1). The complete chloroplast genome of *C. dendroides* can provide a reference for developing markers for further studies on the phylogeny and evolution of the *Climacium* sp. In addition, the cp sequence can be used for species identification or confirmation, which is useful for the conservation and utilization of these multipurpose natural resources.

**Figure 1.**
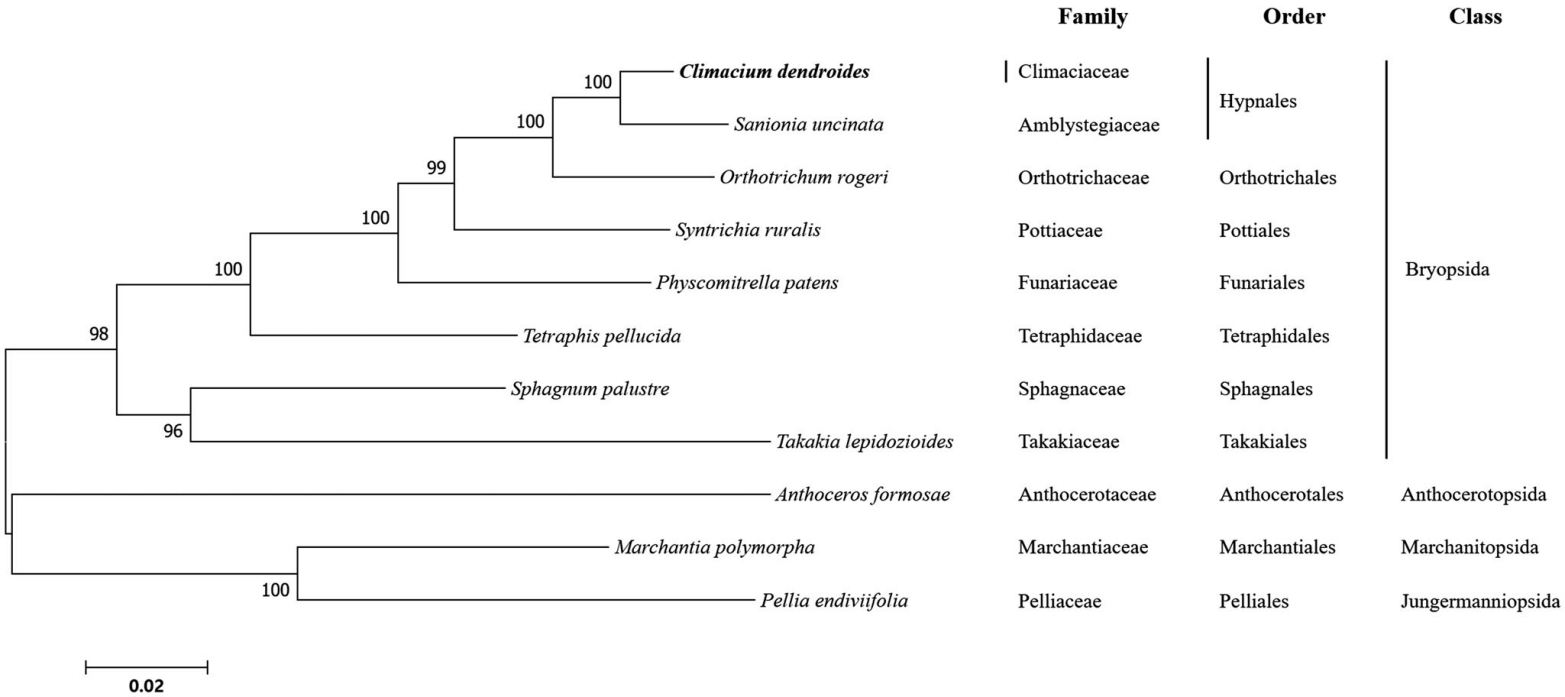
Phylogenetic position of *Climacium dendroides* determined by maximum likelihood analysis based on 16 protein coding genes common in all taxa. The bootstrap values above 50% are indicated at each node. Sequences from liverworts were used as outgroup. GenBank accession numbers of mitogenomes used are *Anthoceros formosae* (NC_004543), *Climacium dendroides* (MT006132), *Marchantia polymorpha* (NC_037507), *Orthotrichum rogeri* (NC_026212), *Pellia endiviifolia* (NC_019628), *Physcomitrella patens* (NC_037465), *Sphagnum palustre* (NC_03019), *Syntrichia filaris* (MK852705), *Takakia lepidozioides* (NC_028738) and *Tetraphis pellucida* (NC_024291).
